# A phyloclimatic study of *Cyclamen*

**DOI:** 10.1186/1471-2148-6-72

**Published:** 2006-09-20

**Authors:** Chris Yesson, Alastair Culham

**Affiliations:** 1 Centre for Plant Diversity and Systematics, Plant Science Laboratories, School of Biological Sciences, The University of Reading, Reading, Berks, England RG6 6AS, UK

## Abstract

**Background:**

The impact of global climate change on plant distribution, speciation and extinction is of current concern. Examining species climatic preferences via bioclimatic niche modelling is a key tool to study this impact. There is an established link between bioclimatic niche models and phylogenetic diversification. A next step is to examine future distribution predictions from a phylogenetic perspective. We present such a study using *Cyclamen *(Myrsinaceae), a group which demonstrates morphological and phenological adaptations to its seasonal Mediterranean-type climate. How will the predicted climate change affect future distribution of this popular genus of garden plants?

**Results:**

We demonstrate phylogenetic structure for some climatic characteristics, and show that most *Cyclamen *have distinct climatic niches, with the exception of several wide-ranging, geographically expansive, species. We reconstruct climate preferences for hypothetical ancestral *Cyclamen*. The ancestral *Cyclamen *lineage has a preference for the seasonal Mediterranean climate characteristic of dry summers and wet winters.

Future bioclimatic niches, based on BIOCLIM and Maxent models, are examined with reference to a future climate scenario for the 2050s. Over the next 50 years we predict a northward shift in the area of climatic suitability, with many areas of current distribution becoming climatically unsuitable. The area of climatic suitability for every *Cyclamen *species is predicted to decrease. For many species, there may be no areas with a suitable climate regardless of dispersal ability, these species are considered to be at high risk of extinction. This risk is examined from a phylogenetic perspective.

**Conclusion:**

Examining bioclimatic niches from a phylogenetic perspective permits novel interpretations of these models. In particular, reconstruction of ancestral niches can provide testable hypothesis about the historical development of lineages. In the future we can expect a northwards shift in climatic suitability for the genus *Cyclamen*. If this proves to be the case then dispersal is the best chance of survival, which seems highly unlikely for ant-dispersed *Cyclamen*. Human-assisted establishment of *Cyclamen *species well outside their native ranges offers hope and could provide the only means of dispersal to potentially suitable future environments. Even without human intervention the phylogenetic perspective demonstrates that major lineages could survive climate change even if many species are lost.

## Background

The prospect of global climate change has directed interest towards investigating the impact of the environment on floral and faunal distribution, speciation and extinction [1,2]. One way to investigate species response to climate is through examination of climatic preferences by constructing bioclimatic niche models (these are also known as species distribution models or environmental niche models) [3-6]. These methods establish preferences of a given species, based on its known distribution, and provide a model of the climate parameters correlating with this. One of the earliest and simplest methods is BIOCLIM, which uses the minimum and maximum (or 95^th ^percentiles) of observed values for each climate parameter to define the environmental niche [3,7]. BIOCLIM's models are more conducive to interpretation than some more complicated methodologies [8], although many comparisons demonstrate that more complex algorithms such as Maxent can have greater predictive value under most conditions [4,9]. Once built, the models can be used in conjunction with different climate scenarios and timeframes to estimate past [10-13], present [9,14,15] and future [2,6,16-18] distributions.

There is an established link between bioclimatic niche models and phylogenetic diversification. Peterson *et al. *[12] suggest that bioclimatic envelopes are heritable and are conserved across evolutionary time. Martinez-Meyer *et al. *[19] demonstrated this using bioclimatic niche models of *Passerina *birds to successfully predict the distribution of sister species. This is further supported by a wider link between climate and phylogenetic diversification [1]. Many researchers are now examining species' climatic preferences across phylogenetic trees [10,20-23]. Of these studies, those concerned with distributions have focussed on present or past distributions [10,20-23]. Yet bioclimatic niche models have also been used to predict future distributions, and their impact on extinction risk [2,6,24]. A clear next step is to examine future distribution predictions from a phylogenetic perspective.

**Figure 1:**
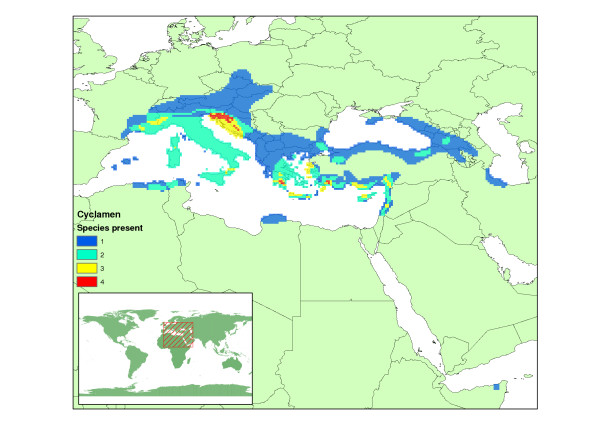
***Cyclamen *diversity**. Species number per 1/4 degree grid square, based on the distribution maps of Grey-Wilson [26].

The genus *Cyclamen *(Myrsinaceae) is a good candidate for such a study, having a well established phylogeny [25], good distribution data [26] and exhibiting adaptations to their seasonal climate [27]. *Cyclamen *are popular garden flowers [26]. They have their own global society of followers in the *Cyclamen *society [28] who have mounted numerous well-documented collection and recording expeditions. *Cyclamen *are primarily distributed around the Mediterranean, but extend eastwards as far as the shore of the Caspian sea [26]. There is also a single isolated species (*C. somalense*) to be found in a small patch of Somalia [29]. Figure 1 shows a complete distribution map for *Cyclamen *based on the maps of Grey-Wilson [26]. Some *Cyclamen*, such as *C. somalense *and *C. libanoticum *have a very limited distribution, and are known from only a few locations. Others such as *C. hederifolium *are widely spread across Europe, even expanding their range into the Southern United Kingdom [30]. The limited dispersal capacity of *Cyclamen *arises from the dependence on ant dispersal of small numbers of large seeds [27], this places them at higher risk from climate change as they cannot disperse easily to newly appearing areas of suitable climate. They are a phenologically interesting group, with at least one species flowering in every month of the year [28], which is unusual for Mediterranean genera which usually show consistent seasonality. The seasonal Mediterranean climate, characterised by hot-dry summers and cooler-wetter winters, is a very important factor for *Cyclamen*. Most *Cyclamen *species remain dormant as tubers during the dry summer months [27]. Current distribution patterns within *Cyclamen *have also been linked to past climate change events [31], prompting the question how could the current predicted climate change affect the potential future distribution of this well-known genus?

Distribution data were gathered for *Cyclamen *from herbarium specimens and distribution maps and these data were used to develop climate profiles for each species. The difference between direct observations and distribution maps was tested and shown to give similar results. BIOCLIM [7] bioclimatic niche models were produced using modelled present day climate data from the Climate Research Unit [32]. These models and underlying climatic parameters were examined from a phylogenetic perspective on the recent, complete species-level phylogeny of Compton *et al. *[25], and a reconstruction of the ancestral *Cyclamen *bioclimatic niche model was performed.

Each species' bioclimatic niche model was examined within a future climate scenario from the Intergovernmental Panel on Climate Change for the 2050s [33]. Future areas of climatic suitability were compared with present day suitable areas and actual distribution. Many species show no future area of climatic suitability and these are considered to be at high risk of extinction due to climate change. This extinction risk measure is examined from a phylogenetic perspective. For full details of methods see methods section below.

## Results

**Figure 2:**
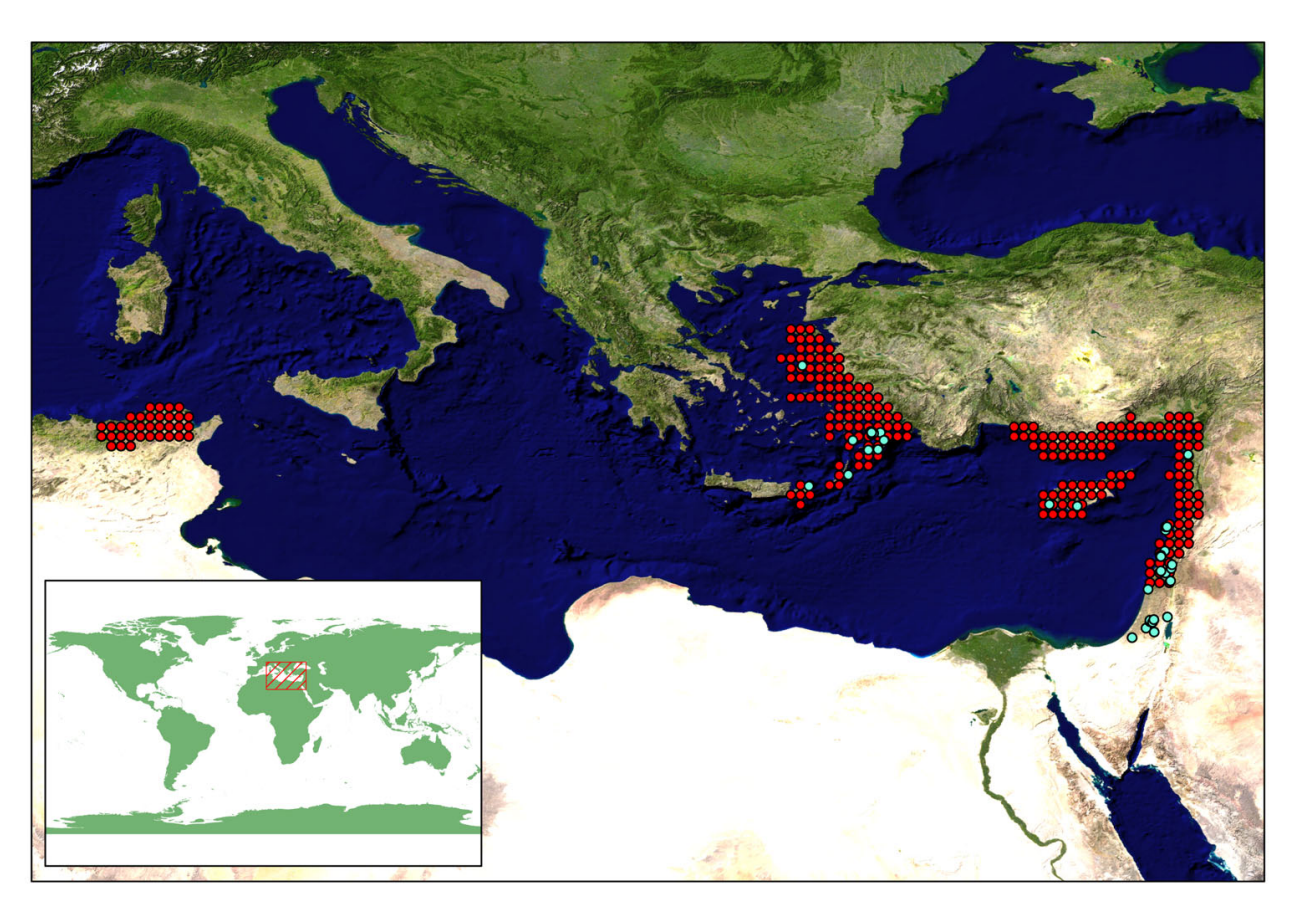
***Cyclamen persicum *distribution data**. Observed point data (pale blue) and points extrapolated from distribution map [26] (red) for *C. persicum. *Blue Marble satellite image.

**Figure 3:**
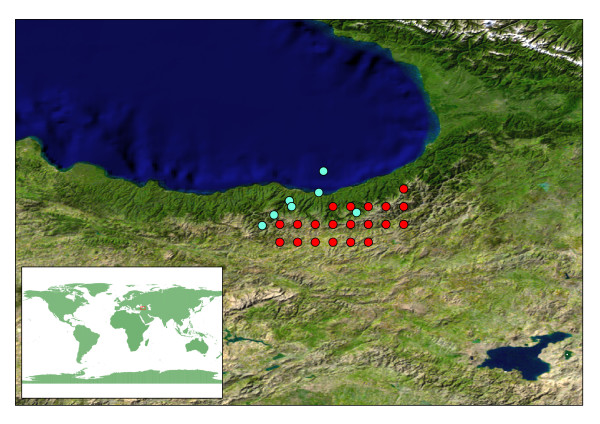
***Cyclamen parviflorum *distribution data**. Observed point data (blue) and points extrapolated from distribution map [26] (red) for *C. parviflorum*. Note: the point with co-ordinates in the Black Sea is excluded from the analysis, and is an example of an excluded point due to poor geographic resolution. Blue Marble satellite image.

When constructing bioclimatic niche models, many authors suggest using direct observations of species locality to construct the model, either via direct collection or through examination of natural history collection data [5,34]. However, sufficient direct observation data is not always available, and in these circumstances good quality distributional data can be used to create "pseudo" observations [20]. For *Cyclamen*, 13 of the 21 species have good direct observations of locality data (min. 20, max. 1,587 points), sourced from herbarium specimen labels or *Cyclamen *Society collecting trips. The remaining species have fewer than 5 observations each, three have none at all. However, all 21 *Cyclamen *species have good extent of occurrence data available via the distribution maps of Grey-Wilson [26]. There is good agreement geographically between the observed data and the distribution maps (some examples are shown in fig 2, 3). The distribution maps encompass most of the directly observed points, but also cover areas for which no observed data are available. For example, *C. persicum *has no direct observations for Tunisia in our locality data sets, but is known to occur in this region (fig 2). In contrast, the directly observed points that occur outside the known distribution are near enough to the known areas to be accounted for by errors of geographic resolution. For example, examining the data for *C. parviflorum *as a worst case scenario (fig 3), we find the point in the sea is as far from land as any other observed point is from the known distribution. There are a few exceptions to this such as the central Israeli distribution for *C. persicum *(fig 2).

**Figure 4:**
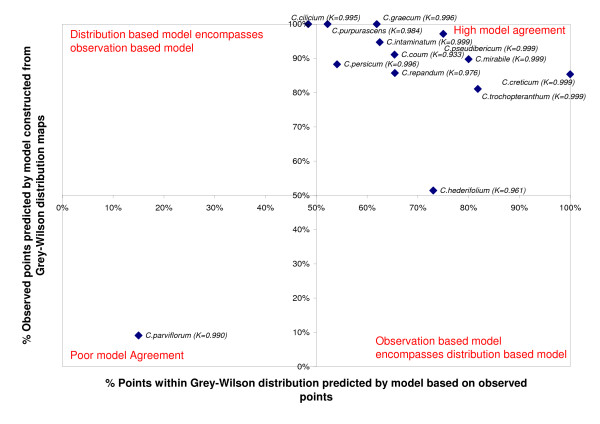
**Similarity of bioclimatic niche models**. Similarity defined as the proportion of real observed points predicted by the model based on distribution maps and vice versa. Comparison plotted for the 13 species with sufficient observed data. K = Kappa statistic of similarity.

Bioclimatic niche models were built using both direct observation data and those gained from distribution maps. The resulting models were compared for the 13 species with sufficient data. Figure 4 shows the results of this comparison. The models demonstrate a high level of similarity, directly comparing the selected envelopes using kappa values gives a minimum of 0.96. If we use a cross projection method then all but two of the models built from the distribution maps predict more than 80% of the observed points. The observed-data models are less efficient at predicting the full distribution, but this is because the observed points do not fully cover all the known areas (e.g. Tunisia for *C. persicum*). For the two outlying species, it is noted that *C. hederifolium *is a widespread species which has an established non-native range as far northwards as the UK. The one species for which the models perform poorly is *C. parviflorum*, it is noted that this is also the species with the fewest observed localities.

The models built via the distribution (area) maps of Grey-Wilson [26] provide a good alternative to those built from direct (point) observations, they also provide the opportunity to study models for all 21 species of *Cyclamen *in a consistent fashion. If there is any bias, it is likely to be due to the distribution maps encompassing an area greater than the true range, which will have the affect of widening the bioclimatic envelope [16]. However, the high agreement of point and area data suggest this effect is minimal, and all subsequent analyses are performed using models built from distribution maps.

**Figure 5:**
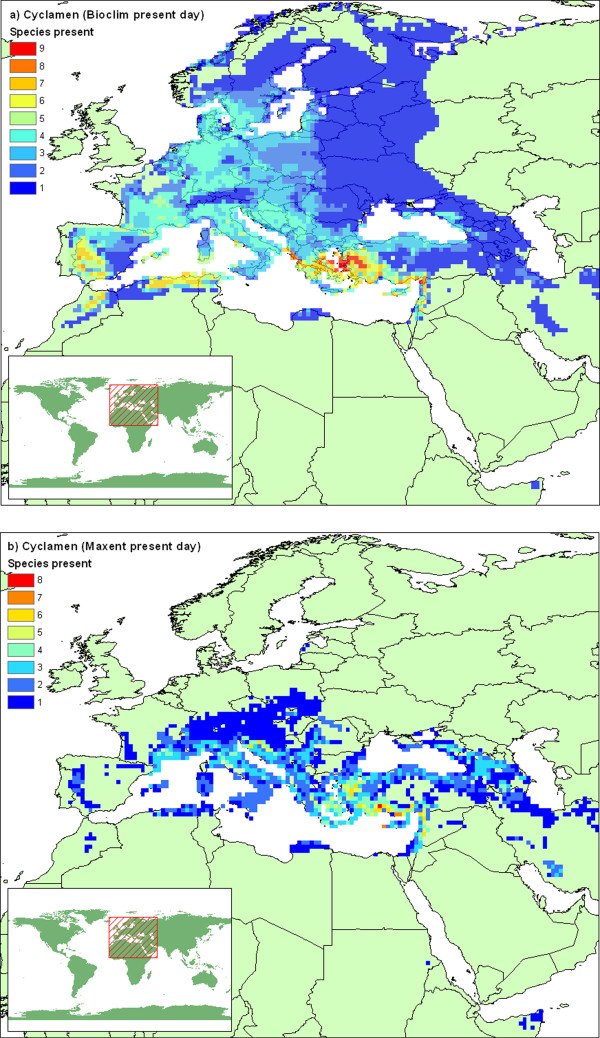
**Modelled *Cyclamen *diversity**. Species diversity mapped on 1/4 degree grid squares, generated by cumulative overlaying climatically of suitable areas for individual species defined by a) BIOCLIM and b) Maxent models.

The bioclimatic niche models give us estimates of the climatic range for each *Cyclamen *species. The Maxent models more closely reflect the original distribution maps (kappa: mean = 0.484, min = 0.067, max = 0.800) than the BIOCLIM models (kappa: mean = 0.430, min = 0.052, max = 0.897). By cumulatively overlaying the areas selected for each species, we get a genus-wide picture of the climatic tolerance of *Cyclamen*. A map of this is displayed in figure 5a, b. These maps can be contrasted with the current distribution displayed in figure 1. The climatic tolerance according to the BIOCLIM models is much wider than the achieved distribution, particularly in the northward direction, in contrast the Maxent models closely mirror the current distribution. The distribution map shows that Greece and Western Turkey are a centre of diversity for *Cyclamen*. The models loosely conform to this pattern. It is noted that the red "hotspot" areas for the distribution map contain 4 species compared with 8–9 for the climatic tolerance hotspots. This shows that several *Cyclamen *species are not present everywhere that is climatically suitable for them.

**Figure 6:**
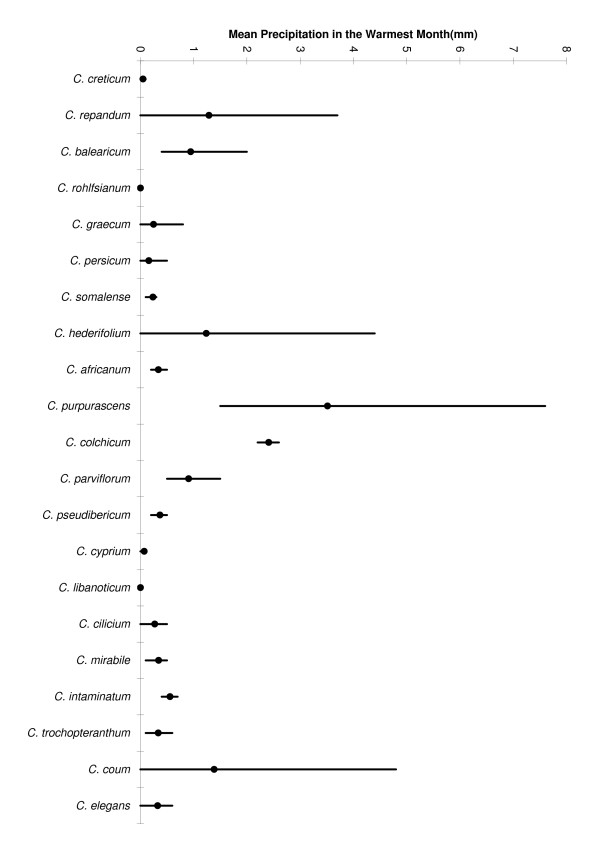
**Range of summer precipitation**. Range of the climatic variable: mean daily precipitation in the warmest month, for all *Cyclamen *species. (Dot indicates species mean, species order follows phylogenetic tree).

Focussing on individual species, we can see a detailed breakdown of the individual climatic layers for each species in table 1 and figure 6. This demonstrates the wide climatic tolerance within *Cyclamen*. The classic Mediterranean-type climate pattern of hot-dry summer (fig 6) and cool-wet winter is evident for species such as *C. cyprium *and *C. libanoticum*, in fact all but 5 species average less than 1 mm of rain in the warmest month of the year. In contrast, there are several species with a wide range of tolerance, for example *C. coum *grows in places with summer rainfall ranging from low to high (fig 6). Species such as *C. rohlfsianum, C. graecum, C. persicum *and *C. somalense *all tolerate highest temperatures above 30 degrees in the warmest month, in contrast species such as *C. purpurascens, C. colchicum *and *C. parviflorum *surviving freezing temperatures in the coolest month (table 1).

**Table 1:** *Cyclamen *climate preferences.

***Cyclamen *Species**	Dev Tmp (0.36)*	**Pcp Wet Mnth (0.68)**	**Tmp Warm Mnth (0.44)**	Mean Tmp (0.36)*	**Dev Pcp (0.60)**	Num Mnth Tmp >0 (0.37)*	Tmp Cool Mnth (0.34)*	Pcp Warm Mnth (0.26)*	Tmp Range (0.37)*	Pcp Dry Mnth (0.35)*	Min Tmp Cool Mnth (0.34)*	**Pcp Cool Mnth (0.61)**	**Mean Daily Pcp (0.52)**	**Hi Tmp Warm Mnth (0.41)**
*C. creticum *(Dörfl.) Hildebr.	5.50	4.33	25.19	17.60	1.58	12.00	10.67	0.05	22.07	0.00	7.35	4.11	1.80	29.42
*C. repandum *Sm.	6.21	3.67	21.96	13.18	0.77	11.10	5.24	1.29	25.13	1.15	1.95	2.54	2.35	27.08
*C. balearicum *Willk.	5.90	2.97	22.81	14.15	0.57	11.54	6.92	0.94	25.38	0.81	2.96	1.91	1.92	28.34

*C. rohlfsianum *Aschers.	5.25	2.33	25.23	18.99	0.89	12.00	11.40	0.00	24.72	0.00	6.72	2.33	0.87	31.44
*C. graecum *Link	6.32	3.97	24.83	15.98	1.35	11.83	7.83	0.25	26.59	0.21	4.11	3.43	1.77	30.69
*C. persicum *Mill.	6.51	4.06	26.11	17.11	1.42	11.86	8.65	0.16	27.73	0.13	4.53	3.73	1.75	32.26
*C. somalense *Thulin & Warfa	3.53	0.83	30.51	26.12	0.22	12.00	21.66	0.23	21.07	0.10	16.00	0.10	0.35	37.07

*C. hederifolium *Ait.	6.54	3.59	21.89	12.81	0.81	10.63	4.22	1.24	26.64	1.08	0.82	2.59	2.23	27.46
*C. africanum *Boiss. & Reut.	6.42	2.71	24.63	15.13	0.76	12.00	7.37	0.34	28.93	0.29	2.92	2.10	1.56	31.84
*C. purpurascens *Mill.	7.06	4.18	16.61	7.38	0.66	7.63	-2.52	3.51	27.63	2.17	-5.72	2.35	3.03	21.92
*C. colchicum *Alboff	9.30	3.64	17.96	6.04	0.73	6.08	-7.79	2.41	37.37	1.32	-12.82	1.35	2.10	24.55

*C. parviflorum *Pobedimova	8.90	2.60	17.29	5.72	0.56	6.05	-7.23	0.91	36.72	0.80	-11.58	1.39	1.57	25.14
*C. pseudibericum *Hildebr.	7.49	4.02	26.65	16.72	1.32	11.33	6.25	0.37	30.50	0.34	2.15	3.72	2.12	32.65
*C. cyprium *Kotschy	6.31	3.36	27.17	18.67	1.23	12.00	10.43	0.07	26.77	0.04	6.26	3.18	1.33	33.03
*C. libanoticum *Hildebr.	6.54	4.50	22.00	13.56	1.73	10.50	4.75	0.00	30.60	0.00	-0.10	4.50	1.73	30.50

*C. cilicium *Boiss. & Heldr.	7.29	3.73	23.45	13.47	1.26	9.98	3.60	0.27	30.62	0.22	-0.55	3.59	1.74	30.07
*C. mirabile *Hildebr.	7.27	5.38	24.63	14.43	1.82	10.88	4.99	0.34	31.42	0.28	0.68	4.97	2.22	32.10
*C. intaminatum *(Meikle) C. Grey-Wilson	6.94	3.33	19.45	10.21	0.92	8.38	0.37	0.55	30.35	0.51	-3.46	2.90	1.85	26.89

*C. trochopteranthum *Schwarz	7.11	4.37	23.82	13.91	1.48	10.41	4.57	0.33	31.33	0.27	-0.01	4.26	1.88	31.32
*C. coum *Mill.	8.04	3.25	20.30	9.71	0.75	8.40	-1.74	1.38	32.46	1.01	-5.74	2.24	1.97	26.73
*C. elegans *Boiss. & Buhse	9.61	1.69	24.57	11.63	0.49	8.32	-2.20	0.32	39.24	0.25	-7.00	1.12	0.92	32.24

Mean values for climatic variables in *Cyclamen *based on distribution data. Species order follows phylogenetic tree. Precipitation is given in mm per day, Temperature in °C. QVI given in parentheses, * indicates QVI is lower than 95^th ^percentile of randomisation replicates.

Key to column headings: Dev Tmp = Standard deviation of mean temperature; Pcp Wet Mnth = Mean daily precipitation in the wettest month; Tmp Warm Mnth = Mean temperature in the warmest month; Mean Tmp = Annual mean temperature; Dev Pcp = Standard deviation of mean daily precipitation; Num Mnth Tmp > 0 = Number of months with minimum temperature above freezing; Tmp Cool Mnth = Mean temperature in the coolest month; Pcp Warm Mnth = Mean daily precipitation in the warmest month; Tmp Range = Annual temperature range; Pcp Dry Mnth = Mean daily precipitation in the driest month; Min Tmp Cool Mnth = Minimum temperature in the coolest month; Pcp Cool Mnth = Mean daily precipitation in the coolest month; Mean Daily Pcp= Mean daily precipitation; Hi Tmp Warm Mnth = Highest temperature in the warmest month.

The substantial difference in winter minimum temperatures (-8 to +21°C) is indicative of the contrast between both inland vs. coastal distributions and latitudinal/altitudinal variation. *C. somalense *is substantially nearer the equator than any other species and experiences warm summers and winters, whilst *C. purpurascens, *the most northerly species, tolerates freezing winter temperatures for at least four months of the year. *C. creticum*, an island species with its climate controlled by proximity to the sea, experiences one of the lowest annual temperature ranges with a mean summer temperature of +25°C compared with +11°C in the coldest winter month. *C. colchicum*, a species growing in continental Asia, experiences one of the highest annual temperature ranges with a mean summer temperature of +18°C compared with -8°C in the coldest month.

### Phylogenetic structure

**Figure 7:**
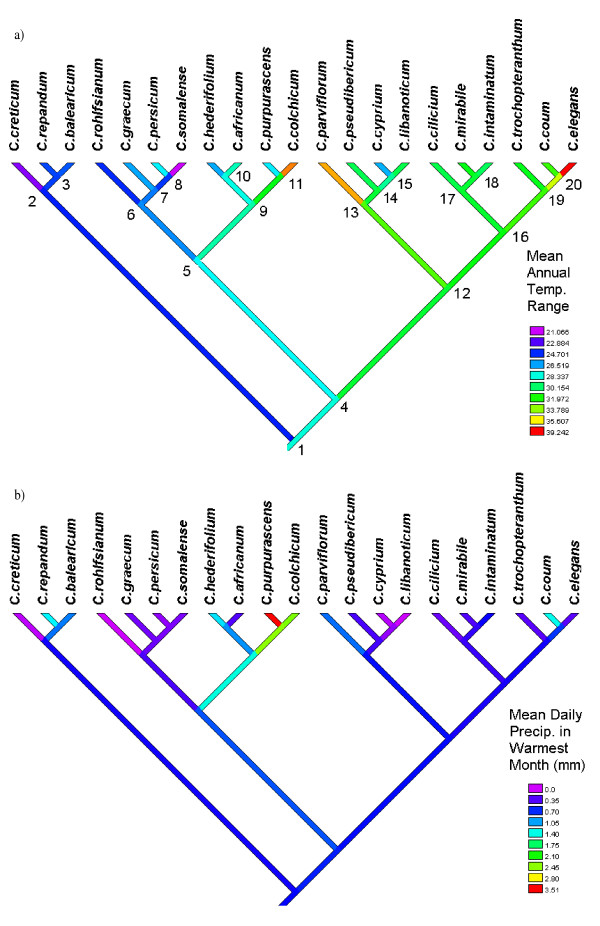
**Phylogeny of *Cyclamen***. Phylogeny of *Cyclamen *with square change parsimony optimisation of a) Mean Annual Temperature Range (°C), performed in Mesquite [59] (QVI = 0.37)*. b) Mean daily precipitation in the warmest month (QVI = 0.26)*. Internal node numbers marked for reference. * Both variables show QVI significantly different from random.

There is some phylogenetic structure to this data, 8 of the 14 climate parameters demonstrate phylogenetic conservancy using the randomisation test of the quantitative convergence index. Figure 7 shows the mean values of the climatic parameter Annual Temperature Range, plotted on the phylogeny of Compton *et al. *[25]. It is evident that the clade comprising *C. repandum, C. balearicum *and *C. creticum *(Subgenus *Psilanthum*) all share low temperature ranges, whilst the clade *C. parviflorum-C. elegans *share higher ranges. In contrast, other climate parameters appear similar for much of the genus, all but 5 species share a dry season with the driest month providing less than 1 mm of rainfall per day. However the phylogenetic structure in the data is not uniform across the tree; for example, comparing *C. elegans *with its wide-ranging sister taxon, *C. coum*, shows that they differ for most precipitation values. Overall, the climate variable mean precipitation in the warmest month has the best fit for this phylogeny (QVI = 0.27). This may, in part, be due to the high level of similarity of this value amongst most *Cyclamen*, also the two highest rainfall values (*C. purpurascens *and *C. colchicum*) are sister taxa.

The phylogeny in figure 7 shows ancestral state reconstruction for annual temperature range. The lineage leading to subgenus *Psilanthum *shares the low range exhibited by its constituent species (fig 7 node 2). Table 2 shows the ancestral reconstruction for the means of all the climatic parameters. The ancestral *Cyclamen *lineage (node 1) has the Mediterranean climatic characteristic of dry summers and wet winters, demonstrated by mean daily precipitation 5 times lower in the warmest month than in the coldest. There is also some frost tolerance exhibited, averaging more than 1 month with minimum temperature below freezing.

**Table 2:** Ancestral reconstruction of climate preference.

**Node**	**Dev Tmp**	**Pcp Wet Mnth**	**Tmp Warm Mnth**	**Mean Tmp**	**Dev Pcp**	**Num Mnth Tmp >0**	**Tmp Cool Mnth**	**Pcp Warm Mnth**	**Tmp Range**	**Pcp Dry Mnth**	**Min Tmp Cool Mnth**	**Pcp Cool Mnth**	**Mean Daily Pcp**	**Hi Tmp Warm Mnth**
node 2	6.35	3.63	23.31	14.64	1.10	10.81	6.23	0.60	26.49	0.46	2.41	3.01	1.82	28.90
node 3	5.96	3.81	23.79	15.51	1.17	11.39	7.86	0.52	24.53	0.42	4.27	3.23	1.89	28.80
node 5	6.02	3.48	22.85	14.28	0.84	11.34	6.67	0.92	25.01	0.79	3.06	2.56	2.05	28.07
node 8	6.75	3.45	22.84	13.78	1.02	10.24	4.61	0.68	28.46	0.50	0.54	2.79	1.75	29.00
node 9	6.53	3.27	22.95	14.27	0.94	10.42	5.34	0.82	27.95	0.56	1.17	2.53	1.70	29.12
node 10	5.87	2.93	24.60	17.01	0.97	11.37	8.94	0.37	26.19	0.26	4.57	2.52	1.33	30.75
node 12	5.83	3.20	25.61	17.77	1.07	11.68	10.08	0.28	25.89	0.20	5.82	2.71	1.42	31.71
node 14	5.29	2.70	27.41	20.34	0.90	11.85	13.46	0.22	24.89	0.14	8.78	2.18	1.17	33.68
node 17	6.99	3.42	21.42	12.03	0.83	9.66	2.47	1.42	29.20	0.94	-1.61	2.28	2.00	27.59
node 18	6.65	3.24	22.65	13.32	0.80	10.76	4.69	1.00	28.26	0.77	0.71	2.32	1.93	28.96
node 21	7.78	3.74	18.67	8.48	0.74	7.79	-2.61	2.45	31.40	1.47	-6.72	1.99	2.38	24.69
node 24	7.35	3.45	22.25	12.41	1.03	9.49	2.26	0.61	30.94	0.48	-1.94	2.83	1.74	29.00
node 25	7.87	3.25	21.21	10.83	0.94	8.61	-0.28	0.63	32.86	0.54	-4.55	2.50	1.71	28.31
node 27	7.37	3.71	24.09	14.36	1.22	10.29	4.14	0.38	30.93	0.33	-0.13	3.29	1.82	30.80
node 29	6.74	3.86	24.42	15.53	1.39	10.93	6.44	0.15	29.44	0.13	2.01	3.66	1.63	31.44
node 32	7.44	3.64	22.71	12.64	1.14	9.61	2.45	0.51	31.49	0.41	-1.82	3.19	1.77	29.67
node 33	7.30	3.85	22.82	12.87	1.24	9.75	2.94	0.41	31.01	0.33	-1.23	3.53	1.82	29.78
node 35	7.17	4.19	22.30	12.51	1.33	9.67	2.77	0.43	30.93	0.37	-1.34	3.80	1.96	29.59
node 38	7.66	3.62	23.06	12.62	1.14	9.60	2.14	0.53	32.52	0.41	-2.28	3.21	1.73	30.24
node 40	8.44	2.85	22.64	11.32	0.79	8.77	-0.60	0.75	34.74	0.56	-5.00	2.19	1.54	29.74

Mean values for climatic variables for internal nodes of the *Cyclamen *phylogeny. Precipitation is given in mm per day, Temperature in °C. Node numbers as fig 7. Column headings as table 1.

**Figure 8:**
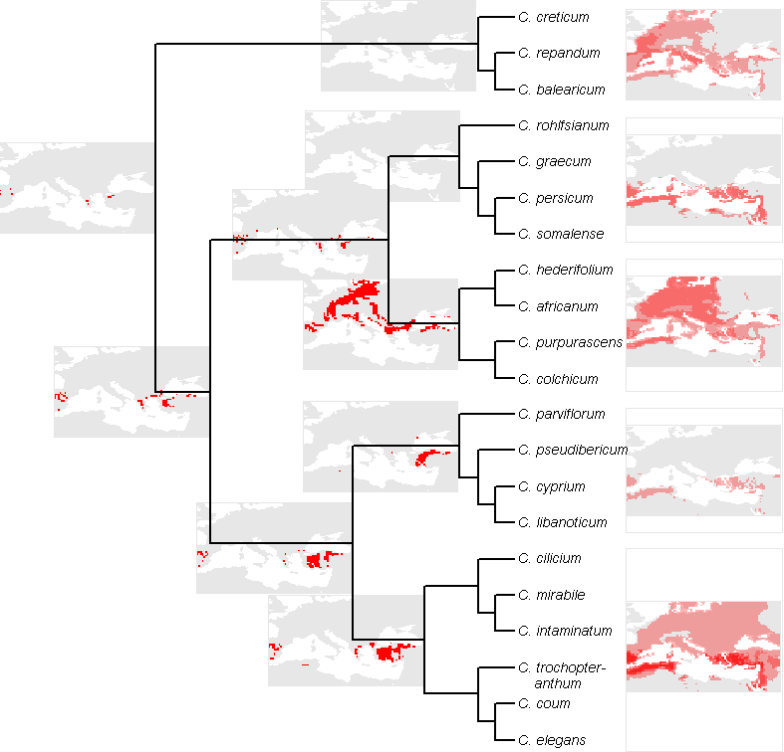
**Reconstruction of bioclimatic envelopes for ancestral *Cyclamen***. Ancestral reconstruction of bioclimatic envelopes examined within present day climate. Right edge of maps are flush with the correct position in the phylogeny. Red areas indicate suitable climate. Models for *C. creticum-C. balearicum *and *C. rohlfsianum-C. somalense *are omitted as they fail to select any area in the present day climate. Models for other internal nodes closer to extant taxa are excluded due to space constraints.

If we examine the ancestral model with reference to today's climate (fig 8), an area of central Greece and Western Turkey is selected which is close to the current centre of diversity highlighted in figure 5. Other internal nodes on the phylogeny each show differing present-day realisations of model reconstructions. For example the lineage leading to the clade comprising *C. hederifolium *– *C. colchicum *demonstrates the broad tolerance of its wider-ranging constituent species. However, there is a predominance of Turkish areas for the majority of these models.

We can examine which of the extant species of *Cyclamen *is closest in climatic preference to our model reconstruction. It is not possible to use the full interpredictivity measure to determine which of the present day *Cyclamen *is most like the ancestral lineage. However, in the area selected by the model you can find the species *C. hederifolium*, *C. repandum*, *C. coum*, *C. graecum *and *C. intaminatum*. No extant species has a distribution similar to this area (Kappa < 0.02 for all species). However, a crude measure of model similarity based on the sum of squares of the differences of means for each climate layer suggests *C. balearicum *is overall the most similar to our ancestral reconstruction.

**Figure 9:**
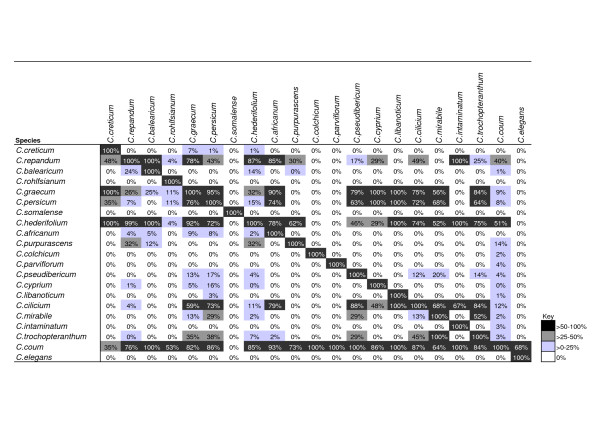
**Cross similarity of bioclimatic niche models amongst *Cyclamen***. Comparison of BIOCLIM models using interpredictivity measure. Heavily shaded cells indicate high agreement. Note the diagonal line indicating 100% self-similarity. Species ordered as phylogeny (fig 7).

As well as comparing the constituent climatic parameters, we can compare directly the resulting BIOCLIM models for each species. Figure 9 displays a measure of model similarity for each pair of *Cyclamen *species. More than two thirds of all comparisons show zero prediction of other species distributions, which suggests that most *Cyclamen *are climatically isolated. It is also clear that there are several climatically wide-ranging species (*C. coum, C. hederifolium, C. cilicium, C. graecum, C. persicum, C. repandum*) which account for the majority (69%) of all positive predictions. The bioclimatic envelope for *C. coum *shows some overlap with the envelopes of all other *Cyclamen *except *C. somalense*.

We can combine sets of these predictions together to produce measures of group similarity, this permits an examination of climatic preferences within a clade. Table 3 shows the mean model similarity amongst the subgenera recognised by Grey-Wilson [26] (Tab. 3a) and selected clades from the *Cyclamen *phylogeny (Tab. 3b). Notice that subgenus *Corticata*, subgenus *Persicum *and the clade above node 13 (*C. parviflorum-C. libanoticum*) all show similarity levels of zero. Several groups (subgenus *Psilanthum *and the clade above node 16) demonstrate mean similarity levels which are almost double that within *Cyclamen *as a whole (Tab. 3c). However, if we test whether these similarity values are above that of a random group of *Cyclamen *of a similar size we find that the group means are not significantly different from the random groups. On closer examination we find that the higher similarity values are driven by the presence of a single wide-ranging species within the groups.

**Table 3:** Similarity of bioclimatic niche models within selected groups of *Cyclamen*.

	**Group (Node No)**	Mean Similarity*	N**	**Std. dev.**	**95% CI**
a)	*Subgen. Corticata *(Node 15)	0.00	2	0.00	-
	*Subgen. Cyclamen *(-)	0.14	6	0.28	0.03–0.23
	*Subgen. Gyrophoebe *(-)	0.22	8	0.35	0.13–0.31
	*Subgen. Persicum *(Node 8)	0.00	2	0.00	-
	*Subgen. Psilanthum *(Node 2)	0.29	3	0.40	0.00–0.60

b)	(Node 6)	0.16	4	0.33	0.00–0.34
	(Node 9)	0.14	4	0.28	0.00–0.30
	(Node 13)	0.00	4	0.00	-
	(Node 16)	0.28	6	0.37	0.15–0.41

c)	All Cyclamen	0.15	21	0.35	0.11–0.18

d)	Random groups of 8 species	0.17	100 × 8	0.07	0.15–0.18
	Random groups of 5 species	0.18	100 × 5	0.10	0.15–0.19
	Random groups of 3 species	0.16	100 × 3	0.25	0.12–0.18

Mean similarity of BIOCLIM models for selected groups of *Cyclamen*. a) " Subgenera; b) Monophyletic groups from phylogeny (fig 7); c) Overall average for all *Cyclamen*; d) Randomisation replicates of subsets of *Cyclamen *for varying group sizes. * Similarity based on interpredictivity measure. ** N is number of species in clade. ***CI = confidence interval for the mean based on N(N-1) comparisons for a-c) and 100 replicates for d)

### Future area projections

**Figure 10:**
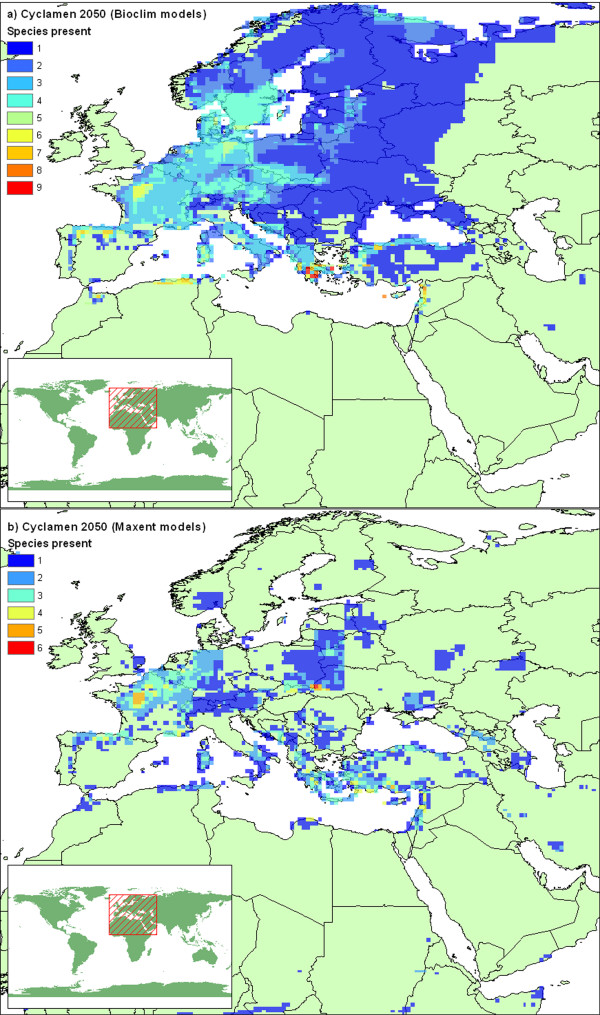
**Predicted future *Cyclamen *diversity with unlimited dispersal**. Predicted species diversity for 2050s mapped on 1/4 degree grid squares (generated as figure 5) from a) BIOCLIM and b) Maxent models examined within a future climate scenario.

**Figure 11:**
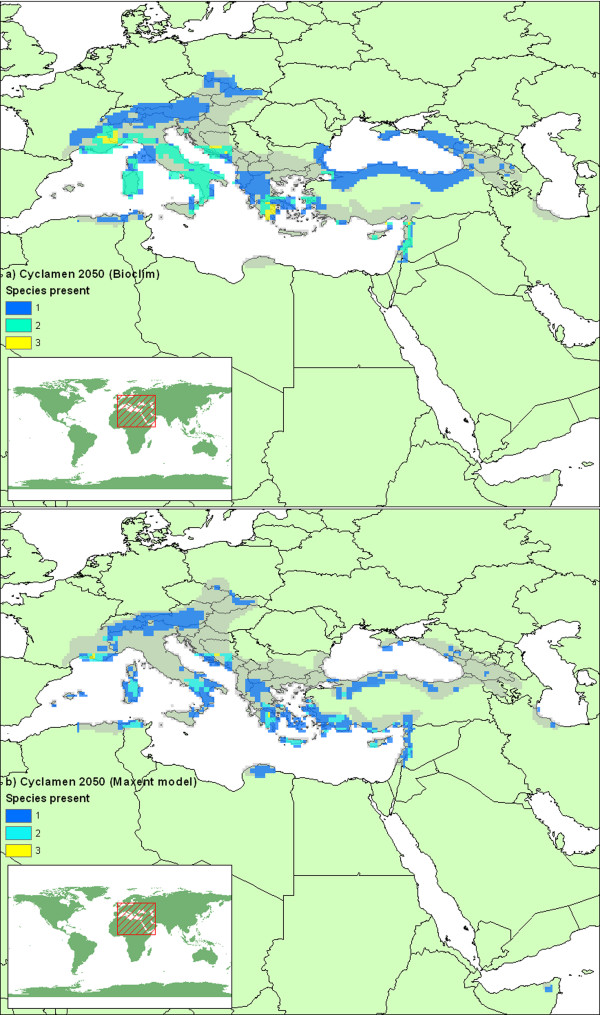
**Predicted future *Cyclamen *diversity with limited dispersal**. Predicted species diversity for 2050s mapped on 1/4 degree grid squares, (generated as figure 5) from a) BIOCLIM and b) Maxent models examined within a future climate scenario. Model predictions are restricted to present day areas and adjacent grid squares under limited dispersal hypothesis. Grey shading indicates present day extent for all species.

The bioclimatic niche models, when examined within a future climate scenario for the 2050s, give an estimate of where each species' preferred climate will be. Figure 10 combines these predictions to repeat the *Cyclamen *diversity map (fig 5) for the future climate scenario. The models with unrestricted dispersal demonstrate a northward shift in the area of climatic preference, which is particularly evident for the Maxent models (fig 10b). The unrestricted dispersal scenario is highly unlikely without substantial human intervention, a more likely scenario is the restricted dispersal hypothesis demonstrated in Figure 11. Here we see major reductions in suitable climate suggested by both algorithms, particularly in central Italy, the former Yugoslavia, Sicily, Southern Turkey and the far eastern extent. Overall, Greece & Turkey still show the most diversity, but the extent is greatly reduced. When we consider the models for each species in turn (Table 4), we find that the area of climatic suitability for every *Cyclamen *has reduced for both modelling algorithms, the majority by more than 60%. Many of these species are considered to be at high risk of extinction due to climate change.

**Table 4:** Occupied and modelled range sizes and extinction risk estimates for *Cyclamen. *Range sizes based on Grey-Wilson's maps [26].

	**Present range**	**Bioclim 2050**			**Maxent 2050**		
**Species**	**(km2)**	**(km2)**	**loss %**	**kappa**	**(km2)**	**loss %**	**kappa**
*C. africanum*	53,365	622	99%	0.385	18,577	65%	0.493
*C. balearicum*	35,690	1,128	97%	0.227	10,503	71%	0.508
*C. cilicium*	59,724	1,873	97%	0.141	14,282	76%	0.545
*C. colchicum*	7,451	0	100%	0.897	1,731	77%	0.326
*C. coum*	453,333	291,502	36%	0.052	77,132	83%	0.352
*C. creticum*	18,936	0	100%	0.816	9,497	50%	0.566
*C. cyprium*	15,796	0	100%	0.518	8,233	48%	0.433
*C. elegans*	28,917	0	100%	0.211	3,692	87%	0.337
*C. graecum*	168,892	71,513	58%	0.222	85,270	50%	0.444
*C. hederifolium*	967,928	473,241	51%	0.321	183,213	81%	0.624
*C. intaminatum*	14,273	0	100%	0.875	2,379	83%	0.528
*C. libanoticum*	2,562	0	100%	0.667	0	100%	0.067
*C. mirabile*	15,321	0	100%	0.254	12,286	20%	0.585
*C. parviflorum*	11,742	0	100%	0.769	2,362	80%	0.437
*C. persicum*	157,919	71,234	55%	0.290	71,535	55%	0.526
*C. pseudibericum*	14,767	1,249	92%	0.226	12,336	16%	0.605
*C. purpurascens*	440,025	232,490	47%	0.255	150,037	66%	0.601
*C. repandum*	513,568	285,493	44%	0.255	67,739	87%	0.625
*C. rohlfsianum*	34,573	0	100%	0.717	22,218	36%	0.800
*C. somalense*	6,797	0	100%	0.720	5,292	22%	0.480
*C. trochopteranthum*	27,769	0	100%	0.204	22,836	18%	0.285

Total	3,049,350	1,430,346	53%	0.430	781,149	74%	0.484

Present day models for BIOCLIM and Maxent algorithms examined within 2050s climate scenarios with restrictions of limited dispersal. Kappa statistic for present day models compared with Grey-Wilson maps.

## Discussion

### Ancestral model reconstruction

Many *Cyclamen *share a preference for seasonal Mediterranean climate characteristics. The ancestral *Cyclamen *was probably well suited to this environment, and may have had a similar climatic preference to the extant species *C. balearicum*. A question remains on the timing of the origin of *Cyclamen *and how this compares with estimates of the emergence of the Mediterranean climate zone.

Although, for some ancestral model reconstructions, no present-day area was within the suitable envelope, this does not invalidate the model, as past climates could exhibit climate types not seen in the present day [35]. However, failure to predict any suitable areas of the relevant palaeoclimate could demonstrate the invalidity of the models. Yesson and Culham [23] have demonstrated the plausibility of this general approach to estimates for ancestral areas based on reconstruction of climate preferences for ancestral lineages in other taxa.

### Phylogenetic signal

There is not a phylogenetic pattern of inherited range size for *Cyclamen*. Several sister pairs of species are seen to have drastically different ranges, all but two species pairs on the phylogeny differ in their total area of distribution by more than 90%. This fits the pattern of schizo-endemic distribution of *C. balearicum *and *C. repandum *found by Thompson *et al. *[36], whereby a single wide ranging species is assumed to be progenitor to a narrow ranging isolated sister species. This is supported by the lack of overlap in bioclimatic niche models. This suggests a combination of geographic, edaphic and climatic constraints are limiting the maximum area of distribution of some species more than others.

**Figure 12:**
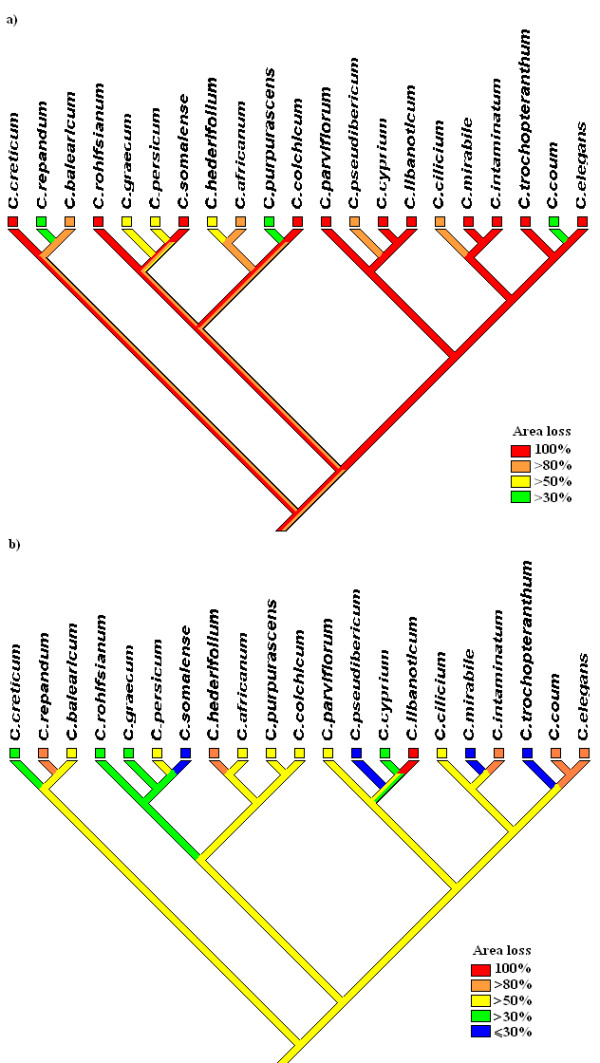
**Parsimony optimisation of extinction risk for *Cyclamen***. Parsimony optimisation of extinction risk based on examination of models within 2050 scenario for a) BIOCLIM and b) Maxent models. Characters treated as ordered, analysis performed in Mesquite

Examining the extinction risk from a phylogenetic perspective (fig 12a, b) we see clearly that within each major lineage there is a pattern of contrasting extinction and survival predictions. BIOCLIM and Maxent predict contrasting patterns of individual species threat. When examined from an overall phylogenetic viewpoint, while many individual species are at high risk, each major lineage is seen to contain at least one species with a reasonable chance of survival. This pattern lowers the overall risk to phylogenetic diversity, and presents the risk in a more favourable light [37,38].

Whilst many of the constituent climatic variables demonstrate phylogenetic conservancy, the resulting models which combine the individual variables into bioclimatic envelopes show little overlap between adjacent nodes. It may be the case that measures for comparing these envelopes are inadequate when the majority of species are climatically isolated from one-another. The only point of comparison of bioclimatic niche models that is currently used is to compare the areas that they select when overlaid into present day data [19,39]. This is also true for the comparison measures used in model validation such as Cohen's Kappa [40]. This is not a direct measure of model similarity. Furthermore such measures may 'hide' real differences for climate types not evident in the present day.

### Climate type

Some studies have found patterns of risk associated with a particular type of climate [18,41]. Thuiller *et al*. [41] found species from Mediterranean climates to be at lower risk of extinction than species from other climates, whereas Thuiller *et al*. [18] found species in Mediterranean regions to be the most sensitive to the changing climate, with species in mountainous regions under great threat but other species having the potential to expand. For *Cyclamen *there is no particular climate-type which is more at risk than another. There is little overlap between any of the bioclimatic niche models of the high-risk *Cyclamen *species, many of these species occur in differing climates and geographies. For example the highest risk group (BIOCLIM models) includes the Mediterranean-type climate of *C. rohlfsianum*; the continental climate of the mountain dwelling *C. colchicum*; the island endemic *C. cyprium*; and the African *C. somalense *which is barely 10° north of the equator. However, for the Maxent models the wide-ranging, more generalist species are consistently assigned a high risk.

### Measures of risk

These broad classifications of risk can hide some noteworthy detail. For example, although *C. balearicum *is designated Endangered (>50% area loss), it is noted that the BIOCLIM future area selection gives no suitable area for this species within the Balearics, and less than 5% of its present day French distribution is considered climatically suitable, indeed most of the predicted suitable climate occurs in Northern France. It is interesting to note that the Balearics are still suitable for other *Cyclamen *species. It is reassuring to note that the French populations of *C. balearicum *have a far higher genetic diversity than the Balearic populations, but the latter demonstrate the higher ecological diversity [31].

There is a link between current range size and extinction risk due to climate change. For the BIOCLIM models every species with a current distribution of under 35,000 km^2 ^is considered the highest risk with the exception of *C. pseudibericum*. In contrast no species with a range size above 35,000 km^2 ^loses all area. Range size and proportion of area lost is strongly negatively correlated for the BIOCLIM (Pearson correlation = -0.816, P < 0.001). In contrast the Maxent models show weak positive correlation of proportion loss and range size (Pearson correlation = 0.332, P = 0.142). The changes in range size show a distinct scattering across the cladograms whether modelled with BIOCLIM or Maxent (fig 12).

### Methodological issues

There are of course other factors than climate limiting species distributions. At the fine scale factors such as soil, aspect, and surrounding vegetation will have a role in determining the establishment of individuals given an otherwise suitable climate. Such fine-scale limiting factors are of secondary importance when modelling species distribution at a global or regional scale [42]. These criticisms of species distribution modelling and the extinction risk measures based on them are discussed at length in other places [6,19,41-47] and we refer the reader to these references.

The BIOCLIM models are restrictive in their definitions of the edge of a suitable climate, predictions for all areas are essentially a binary presence/absence and do not allow any climatic parameter to be outside the observed range [47]. For example, the BIOCLIM models predict no *Cyclamen *in the UK, but we know that three species are naturalised there. Alternative modelling algorithms such as Maxent have the advantage of producing probability measures rather than binary presence/absence, which provide more realistic edges to ranges [48]. However, for the UK naturalised *Cyclamen*, the Maxent models also fail to select any of their naturalised distribution within the selected threshold.

Both modelling algorithms predict large range loss for all *Cyclamen *species, and the predicted future diversity maps are broadly similar (fig 11). However, predictions for individual species differ dramatically. The Maxent models show the highest area loss, yet the BIOCLIM models suggest many more species at the highest extinction risk. This kind of algorithm dependent difference has been observed in other studies [44,49].

For the BIOCLIM predictions, it is evident that range size is a key factor in determining extinction risk due to climate change. Those species at high risk tend to have smaller distributions and smaller climatically suitable areas. This is in general agreement with others who have found a relationship between niche breadth and projected range loss [18,41], (it is trivial to note that for *Cyclamen *the widest-ranging species have the broadest niches). However, the relationship between range size and range loss reported by Thuiller *et al*. [41] is a bell shaped curve with the largest ranges suffering substantial losses, although it is possible that no *Cyclamen *would fall into the category of truly wide-ranging. In contrast the Maxent models demonstrate the opposite pattern (albeit weakly), with widest-ranging species losing proportionally more area. It should be noted, however, that bioclimatic niche models may be less accurate for wide-ranging generalist species [4,50].

Thuiller *et al. *[51] considered selecting the best algorithm for each species in turn based on validation scores. Such a policy, in this case, would combine the highest area loss of both methods producing high overall area loss and high numbers of species with 100% projected area loss. However it is to be noted that present day validation may not reflect model validity for the future [46,51].

The main advantage of BIOCLIM in the context of Phyloclimatic modelling is its independent treatment of climatic variables. As far as we are aware, this is the only method that permits independent optimisation of environmental characteristics on a phylogeny following established phylogenetic reconstruction methods. These optimised variables can then be directly converted into a BIOCLIM bioclimatic niche models. Reconstruction of ancestral states on a phylogeny requires a distance metric to directly compare the output models of different species. There are many problems associated with using the more complex (and often more precise) models currently being developed. For example, many discard non-informative input parameters, meaning models are not directly comparable amongst species as different species models will be built with different subsets of variables. Other models are based on the amalgamation of multiple model outputs, again making direct comparison of models difficult.

## Conclusion

Examining climate preference envelopes from a phylogenetic perspective brings new insight into this field. Ancestral climate envelope reconstruction can provide testable hypotheses about the development of lineages. In the case of *Cyclamen*, the ancestral lineage most probably developed in a Mediterranean type climate similar to parts of present day Greece and Turkey.

Climate parameters often show phylogenetic conservancy within *Cyclamen. *Despite this, the resulting bioclimatic envelopes show startling contrast between many sister species. The consequence is that while individual species are often at high risk, extinction is not predicted for most major lineages. The pattern of contrasting niches may not always be the case and it is likely that in groups demonstrating clearer patterns of niche conservatism it may be possible to assign risk of area loss due to a changing climate by phylogenetic proximity. This approach may be useful where data for full scale niche modelling is not available.

Many *Cyclamen *species are at high risk from the changing climate, and none will remain unaffected. Over the next fifty years we can expect a northwards shift in climatic suitability for the genus *Cyclamen*. Many species face the prospect of their local climate changing so much that their current distribution will be outside their current observed climate tolerance. If this proves to be the case then survival requires either adapting over a very short timeframe (implying exaptive changes or potentially suicidal rates of natural selection) or dispersing to new areas. The long distance migration option seems highly unlikely for ant-dispersed *Cyclamen*. This is worsened by the presence of many geographic barriers, the largest being the Mediterranean sea. Although all *Cyclamen *are listed in CITES, which protects them from trade exploitation from the wild, this does not per-se prevent their extinction though habitat loss by climate change. There are, however, still reasons for hope, the garden use of several *Cyclamen *species in more northern areas of Europe has facilitated the establishment of some *Cyclamen *species well outside the climatic zone of their native ranges. This suggests at least some climatic exaptation and the presence of a nucleus of plants in an area that will become climatically optimal for *Cyclamen *in the future. This provides a novel means of dispersal to, and colonization of, potentially suitable future environments.

## Methods

### Phylogeny

The 21 species recognised by Compton *et al. *[25] were used for this study. Their phylogenetic study of *Cyclamen *is used to define relationships within *Cyclamen *for this paper. Their study produced phylogenies based upon nuclear (ITS) and plastid (*trn*L-F) DNA, as well as morphological data. The phylogenetic trees have strong topological similarity, differing mainly in degree of resolution. One of the two maximum parsimony trees based on the combined ITS + *trn*L-F dataset is used throughout this analysis. The alternative topology differs only in the position of *C. graecum *relative to *C. rohlfsianum *within series *Persicum*, our chosen topology agrees with findings of Clennett [52] with regards to this clade.

### Locality data

Locality data were collected for each of the 21 *Cyclamen *species. These data came from four sources. Firstly from *Cyclamen *Society collecting trips, which has made many trips over the period 1987–2004 covering Greece and the Greek islands, Israel, Sardinia and Turkey, providing 2,315 observations for 12 species [28]. Secondly, data from the Global Biodiversity Information Facility [53], provides 232 observations for 6 species from 6 institutions covering Austria, Croatia, Germany, Greece and the Greek islands, Israel, and Italy (a full list of institutions in provided in the appendix [see Additional file 1]). Thirdly, data from specimen labels at the University of Reading Herbarium (RNG) containing 61 observations for 12 species covering the Balearics, Algeria, Austria, the Caucasus, Corsica, Croatia, Cyprus, Greece and the Greek islands, Israel, Italy and Turkey.

Points were excluded if they fell in an ocean or sea. Exclusions were minimal, comprising fewer than 3% of the total observations, primarily being records from near coastlines with poor geographic resolution (fig 3 shows an example of poor geographic resolution). Species with fewer than 10 observations were excluded from analyses, following Graham *et al. *[20]. Data from the UK National Biodiversity Network for *C. hederifolium, C. repandum *and *C. coum *were excluded from the analysis as these represent records outside the native range of these species [34]. Exclusions reduced the number of analysable *Cyclamen *down to 13 species.

The fourth source for distribution data was the detailed distribution maps of Grey-Wilson [26]. This alternative approach allowed analysis of all *Cyclamen *species. These maps were digitally captured and pseudo distribution points were produced by taking the centroid of every overlapping quarter degree square. Quarter degrees were chosen as this is the resolution of the climate data used in this study. This produced distribution data for all 21 *Cyclamen *species. Due to its restricted distribution *C. libanoticum *has only 5 pseudo-localities following this methodology, and is the only species that falls below the 10 observations recommended by Graham *et al. *[20], however reasonable models were still produced for this species, and this species is included in all analyses.

*Cyclamen *range size was estimated by summing the area of each quarter degree grid square that overlapped with the digitised distribution maps.

### Climate data

Present day observed climate data were taken from an observed climatology dataset (known as CRU CL1) from the Climate Research Unit [32,54]. Future data were taken from the Hadley centre general circulation model (HadCM3) from the IPCC website [33]. The period 2040–2069 hereafter referred to as 2050s was chosen as the future time-frame. This allows sufficient time for the effects of climate change to be detectable, whilst minimising the overall timeframe to reduce uncertainties [55,56] There are many different future climate scenarios based on different predictions of socio-economic factors, we have chosen the A2 scenario representing the mid-range of climate change severity [56]. In order to produce future climate datasets which are directly comparable with the observed data, we applied modelled changes in climate to the present day observed data. The modelled changes were calculated by subtracting the 2050s prediction from directly comparable modelled present day climate following Peterson *et al. *[24] and IPCC recommendations [57].

Both the present and future datasets were processed into biologically meaningful climate profile parameters [7]. Standard deviation of mean temperature, mean daily precipitation in wettest month, mean temperature in warmest month, mean temperature, standard deviation of mean precipitation, number of months with minimum temperature above freezing, mean temperature in coolest month, mean daily precipitation in warmest month, annual temperature range, mean daily precipitation in driest month, lowest temperature in coolest month, mean daily precipitation in coolest month, mean daily precipitation, highest temperature in warmest month. These are similar to Busby [7] and were processed using the "Climate Data Processor" plug-in developed by Tim Sutton as part of the Quantum GIS project [58].

### Modelling

The mean, standard deviation, minimum and maximum values were collected for all climatic parameters, for all species. Each value was independently optimised as a continuous character across the chosen phylogeny, using square change parsimony optimisation implemented in Mesquite [59], following the methodology of Graham *et al. *[20]. A table showing each of these optimised values for every node on the tree is provided in the appendix [see Additional file 2]. These values define a BIOCLIM bioclimatic niche model [3] for each node on the tree. Models were created and manipulated using the BIOCLIM algorithm of the openModeller software package version 0.3.4 [60].

Additional models were constructed using the maximum entropy method (Maxent) for modelling species geographic distributions [9]. This new method has been shown to be very effective for species distribution modelling [4]. Models were built using the same data as the BIOCLIM models using Maxent version 2.3.0 [61]. Thresholds were calculated for models to maximise the Kappa statistic. These thresholds were used to determine presence/absence for present day and future projections.

The quantitative convergence index (QVI) [62] was calculated for each optimised climate parameter. This measure is analogous to 1 minus the retention index for discrete characters. A randomisation test of this value was performed [63]. A random shuffle of the terminal node values was optimised on the chosen topology, this was repeated 100 times for each character. The observed QVI was compared with the distribution of the randomisation replicates to test if the observed value is different from a random placement of data on the fixed phylogeny. If the observed QVI was outside the 95% confidence interval of the randomisation replicates then it was considered significantly different from random [63].

Only the variables demonstrating phylogenetic conservancy, with reference to the QVI, were used in the construction of ancestral bioclimatic niche models. This reduced the number of variables used in this stage of the modelling from 14 to 8.

Similarity of bioclimatic niche models was assessed using interpredictivity calculations [19,39]. This involves the overlaying of locality records of one species (Species A) into the predicted area of another (Species B). Similarity is measured as the percentage of points of A falling within the prediction area of the model of B, and vice versa [19]. Note this is not a symmetric measure, for example, if the envelope for A completely encompasses B then we could have B⊂A but A⊄B, therefore both directions of similarity were performed. Similarity was also assessed using Cohen's Kappa statistic [40,64]. Mean similarity for a group of species was assessed by averaging similarity values of each pair of species within the group.

Summary 'hotspot' maps were produced by creating binary presence/absence maps for each species based on their bioclimatic niche model output, then summing the number of species predicted in each quarter degree cell. This was accomplished using software accessible through the openModeller project [60].

Present day bioclimatic niche models were examined within the 2050s climate scenario to produce a future distribution estimate based on climate suitability. Species with zero area predicted as climatically suitable are selected as high extinction risk [65]. When predicting future distributions and extinction risk it is necessary to take dispersal rates into account [2,66]. Plant migration rates have been estimated at 20–40 km over a 100 year timescale [67], more recently even lower rates have been suggested [68], it is highly unlikely that the ant-dispersed *Cyclamen *will exceed these dispersal rates. Our analysis uses a fifty year timescale and a geographic resolution of approx 25 × 25 km squares, so we employ a model permitting dispersal only within cells immediately adjacent to the current distribution. Therefore species which have future predictions which do not overlap with this the slightly expanded present distribution are also considered at risk, though this is regarded as lower risk than the case where there is no suitable area. Risk categories are assigned according to IUCN classifications based on area loss over 505 years [16,69], namely: Extinction 100% loss, Critical >80%, Endangered >50%, Vulnerable >30%

## Authors' contributions

CY & AC conceived and designed the study, CY conducted the analysis, CY & AC prepared the manuscript.

## Supplementary Material

Additional file 1 Appendix1. Microsoft Excel spreadsheet listing names of institutions providing data for this analysis. Click here for file

Additional file 2 Appendix2. Microsoft Excel spreadsheet listing mean, standard deviation, maximum and minimum values for all climate parameters for extant *Cyclamen *and ancestral state reconstructions. Click here for file
